# Junction Physics and Architectural Paradigms in Optoelectronic Semiconductor Fibers

**DOI:** 10.1002/advs.76017

**Published:** 2026-06-09

**Authors:** Hailiang Wang, Wenshu Ouyang, Yuhang Xiao, Wei Yan, Meifang Zhu

**Affiliations:** ^1^ State Key Laboratory of Advanced Fiber Materials College of Materials Science and Engineering Donghua University Shanghai China

**Keywords:** Electronics, Fabrication, Fibers, Materials science, Semiconductors

## Abstract

Optoelectronic fibers are emerging as a foundational platform for distributed sensing, energy harvesting, optical communication in wearable electronics, soft robotics, and embodied intelligence. Unlike planar optoelectronic devices, fibers must intrinsically integrate light emission, detection, and modulation within scalable, deformable textile networks. In one‐dimensional semiconductor fibers, device performance is ultimately constrained by junction formation under geometrically confined and dynamically evolving processing conditions, yet a unified framework for understanding and designing such systems remains lacking. Here, we establish a systematic junction centric framework that connects semiconductor physics, fiber‐processing constraints, and device performance, thereby enabling coherent analysis and design across fiber solar cells, photodetectors, and light‐emitting diodes. We then clarify how fiber‐confined processing couples with microstructural evolution and junction formation, showing that the thermal, fluidic, and mechanical histories experienced during fabrication govern morphology and crystallization, thereby determining junction quality and device performance. These insights are further distilled into actionable strategies for realizing high fidelity in‐fiber junctions. Finally, we highlight axially continuous and axially segmented junction configurations as key architectures for functional miniaturization and multifunctional integration beyond uniform fiber systems. Looking forward, major opportunities lie in integrating next‐generation semiconductors into fibers, achieving high‐fidelity junctions under continuous manufacturing, and realizing spatially addressable optoelectronic fibers for scalable system level integration.

## Graphical abstract

Optoelectronic fibers are emerging as a key platform for distributed sensing, energy harvesting, and optical communication in deformable systems. Their performance is fundamentally governed by junction formation under confined, dynamic processing conditions. Establishing a junction‐centric understanding is therefore essential for controlling microstructure, ensuring junction fidelity, and enabling high performance fiber based optoelectronic devices.

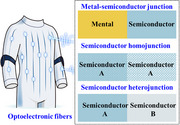

## 1 Introduction

Optoelectronics concerns the generation, detection, and control of light through electronic devices. Its origins trace back to foundational discoveries like the photovoltaic effect (Becquerel, 1839) and the photoelectric effect (Einstein, 1905), with advancements accelerated by key device innovations including the first Cat's Whisker Detector (Ferdinand Braun, 1874), the practical silicon solar cell (Bell Labs, 1954), and the first practical visible‐spectrum light‐emitting diodes (LEDs, Holonyak at GE, 1962). Up to now, silicon‐based devices dominate and enable critical technologies across energy, communications, computing, and healthcare, but their fabrication relies on rigid planar substrates via complex epitaxy and microfabrication, which are inherently incompatible with the emerging demands for miniaturized, flexible, and wearable compatible devices. In comparison, textiles provide an ideal platform for human integrated optoelectronics, offering tunable architectures, conformal body interfaces, mechanical resilience under complex deformations, and scalable manufacturability. Consequently, integrating functional devices directly into textile structures represents a vital pathway for next generation optoelectronics [1, 2, 3, 4, 5, 6, 7, 8, 9].

**FIGURE 1:**
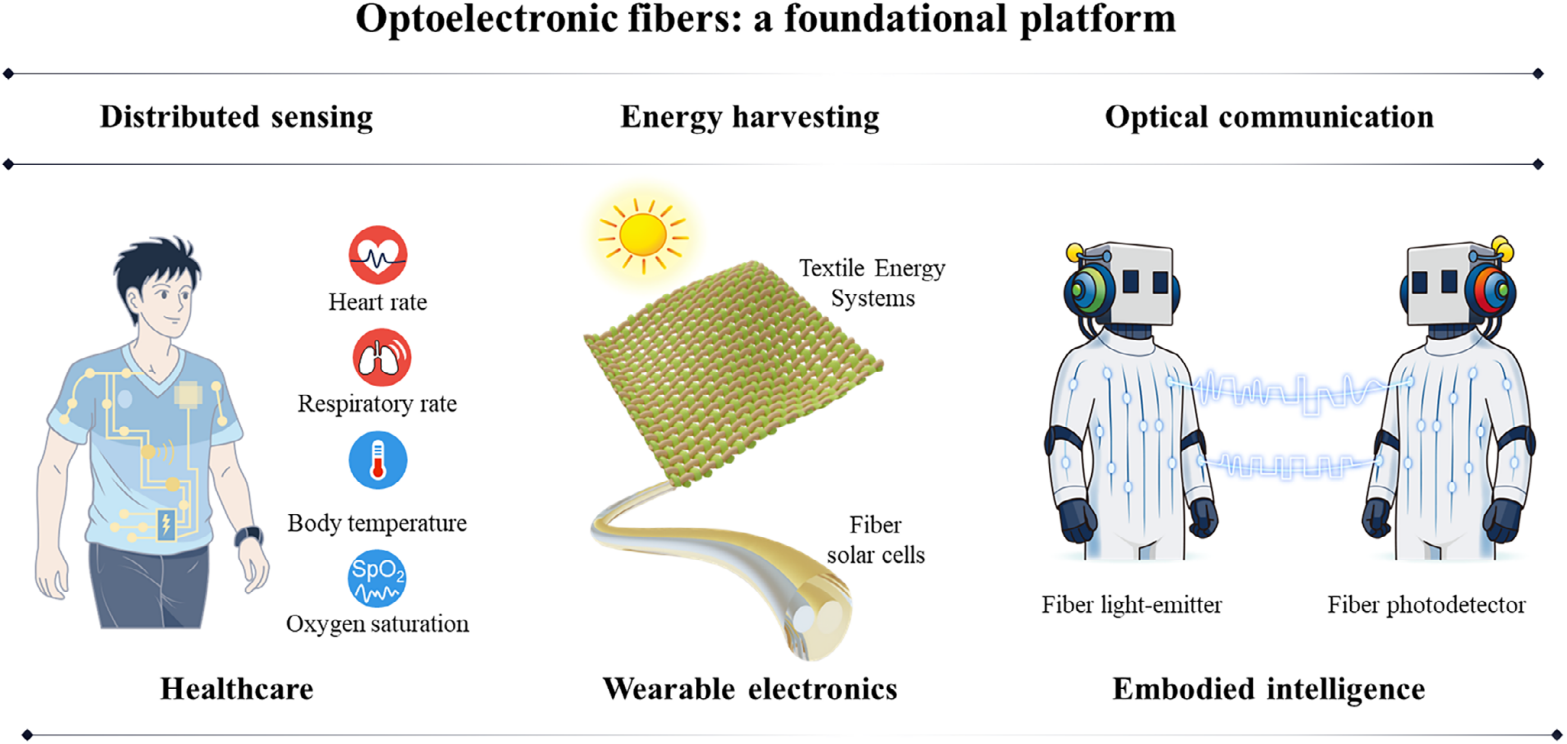
Optoelectronic fibers as a foundational platform for distributed sensing, energy harvesting, and optical communication in wearable electronics, healthcare, and embodied intelligent systems.

Fibers constitute the fundamental structural units of textiles and represent a foundational material morphology. Throughout history, from ancient natural fibers to modern synthetic fibers, high performance fibers, and optical fibers, their development has been driven by continuous multifunctionalization [10, 11]. Optoelectronic fibers are emerging as a foundational platform for distributed sensing, energy harvesting, and optical communication in wearable electronics, healthcare, and embodied intelligence (Figure 1). These encompass energy harvesting units (solar cells), display devices (light‐emitting diodes), and sensors (photodetectors, transistors) [12, 13, 14].

**FIGURE 2:**
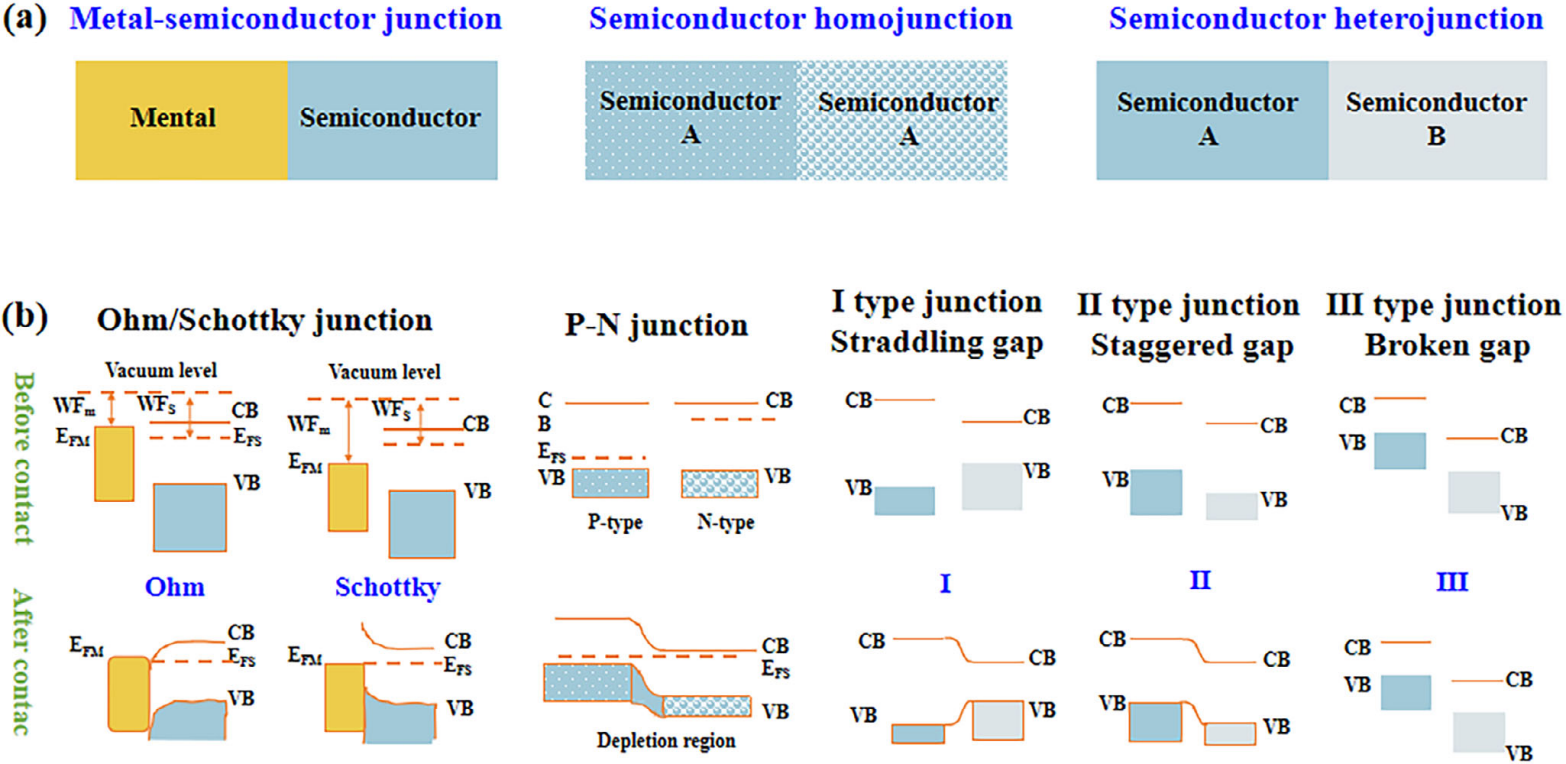
(a) Schematic diagrams of metal–semiconductor contact, semiconductor homo‐junction and semiconductor heterojunction. (b) Schematic diagram of the energy level structure of a typical junction.

High performance optoelectronic fiber devices require high quality semiconductor cores as the functional materials that serve as the fundamental components. Further, most optoelectronic technologies rely on the use of junctions between semiconductors and other materials to enable efficient device operation [13, 14, 15, 16, 17]. The fundamental concept behind these junctions is the combination of two components with different optoelectronic properties. For example, the well‐established silicon (Si) solar cells are based on a different type of junction, one that is formed by p‐ and n‐type doping on opposite sides of a silicon homojunction (Figure 2a) [18, 19]. Thus, in‐fiber semiconductor processing and the realization of in‐fiber junctions are the key to fabricating fiber optoelectronic devices and optimizing their performance.

In this review, we outline strategies for engineering semiconductor materials, junction architectures, and structural geometries within fiber substrates, together with their corresponding fabrication techniques. We begin by introducing the fundamental types of semiconductor junctions and the operating principles of three representative fiber‐integrated optoelectronic devices. We then survey key fabrication approaches for semiconductor fibers, including thermal drawing, solution based coating, and vapor assisted deposition. Subsequent sections discuss the morphological and crystallization control of semiconductor fiber cores to achieve targeted geometries and phases. We further examine the design of in‐fiber junctions and the formation of well‐defined interfaces with complex structural layouts. Finally, we highlight current challenges and outline future research directions that underpin advances in semiconductor fiber technologies.

## 2 Fundamentals of Semiconductor Fiber Devices

### 2.1 Types of Semiconductor Junctions

A semiconductor junction refers to the interface region where two materials or regions with distinct electronic properties meet, including metal–semiconductor junctions, semiconductor homojunctions, and semiconductor heterojunctions (Figure 2a). Its primary function within electronic and optoelectronic devices is to establish a space‐charge region and thus a local built‐in electric field. This engineered asymmetry is essential for controlling the directional flow of charge carriers including electrons and holes, enabling critical device operations such as charge separation, rectification (current flow in one direction), switching, amplification, and sensing. In photovoltaics specifically, the junction's built‐in field provides the driving force necessary to separate photogenerated electron–hole pairs before they recombine, thereby converting light energy into electrical energy.

#### 2.1.1 Metal–Semiconductors Junctions

When a metal and a semiconductor form a contact, the relative magnitudes of their work functions determine whether an Ohmic contact or a Schottky contact is created. Considering an n‐type semiconductor as an example:

Ohmic Contact: As shown in Figure 2b, a low‐resistance junction forms when the metal work function (Φ_m_) is less than the semiconductor work function (Φ_s_) (Φ_m_ < Φ_s_). Upon contact, electrons flow from the metal into the semiconductor. This results in downward band bending near the interface, creating an accumulation region rich in majority carriers (electrons). Consequently, the barrier for electrons to flow from the semiconductor into the metal is drastically reduced. An Ohmic contact therefore allows current to flow easily and linearly in both directions (from the metal to the semiconductor and vice versa), behaving essentially like a small resistor.

Schottky Contact: As shown in Figure 2b, a rectifying junction forms when the metal work function is greater than the semiconductor work function (Φ_m_ > Φ_s_). Upon contact, electrons flow from the semiconductor into the metal. The bands bend upward near the interface, thereby creating a depletion region devoid of majority carriers (electrons). This establishes a significant Schottky barrier (Φ_B_) for electrons attempting to move from the semiconductor into the metal, with Φ_B_ ≈ Φ_m_—χ_s_ (where χ_s_ is the semiconductor's electron affinity). Similar to a p‐n junction diode, a Schottky contact presents high resistance to current flow under reverse bias and low resistance under forward bias.

#### 2.1.2 Semiconductor–Semiconductors Junctions

2.1.2.1

P‐N Homojunction: A p‐n homojunction is formed within a single semiconductor material by creating adjacent regions doped with acceptor impurities (p‐type, hole‐rich) and donor impurities (n‐type, electron‐rich). The resulting band bending at the interface generates the crucial built‐in electric field (Figure 2b). This is the fundamental structure of traditional silicon solar cells and diodes.

2.1.2.2

Heterojunction: A heterojunction is formed by joining two chemically and structurally different semiconductor materials with complementary properties such as light absorption and carrier transport, thereby overcoming some of the limitations of a single material system. The difference in their fundamental properties, particularly their electronic band structures (for example, bandgap and electron affinity), determines the junction's behavior. Heterojunctions are further categorized based on band alignment. Type‐I (straddling) favors confinement of both carriers. Type‐II (staggered) promotes the spatial separation of electrons and holes across the interface, which is highly advantageous for charge separation in photovoltaics. Type‐III (broken‐gap alignment) can enable interfacial tunnelling transport (Figure 2b).

### 2.2 In‐Fiber Fabrication Strategies

#### 2.2.1 Solution‐Based Coating

Solution coating represents the predominant technique for depositing functional materials onto planar or fiber substrates layer by layer, valued for its simplicity and rapid processing. This method is broadly categorized by its film formation mechanism into physical deposition coating and reactive synthesis coating.

Physical deposition coating: This method relies solely on physical processes without chemical reactions or phase transformations of the solute. The fundamental mechanism involves two sequential stages: (1) the adhesion of a solution or dispersion onto the substrate surface during immersion and withdrawal, followed by (2) the evaporation of the volatile solvent, leading to the physical deposition and consolidation of the non‐volatile solute components. The resulting film composition is identical to the initial solute. A representative example is the formation of a conductive layer via dip coating using a carbon nanotube paste, in which nanotubes are deposited primarily through physical accumulation upon solvent evaporation. Likewise, perovskite quantum dots （QDs）or electrolytes are often introduced through multiple coating cycles to construct the functional layers. In this case, layer formation is dominated by physical deposition processes with chemically driven interfacial growth or reconstruction playing only a secondary role [20, 21].

Reactive synthesis coating: This approach extends beyond physical deposition by incorporating chemical reactions or crystallization during or following the coating step. While solution adhesion and solvent evaporation remain initial stages, the precursor subsequently undergoes chemical conversion. This typically involves thermal treatment to drive precursor decomposition or reaction, forming new chemical compounds, and to induce crystallization of the desired functional material. A classic example is the formation of perovskite films, where precursor compounds dissolved in the coating solution react and crystallize into the perovskite structure upon thermal annealing after deposition. For example, lead iodide or tin iodide, together with methylammonium iodide and the required additives, can be dissolved in a single precursor solution, into which the fiber substrate is dipped to deposit the perovskite layer [22, 23].

More complex coating methods can be designed by using multiple precursors and chemical reactions, which may involve sequential coating. In such approaches, the fiber‐shaped core substrate is dipped sequentially into different precursor solutions. In many reported scenarios, a two‐step sequential dip‐coating process has been proven to be effective in forming a good‐quality CH_3_NH_3_PbI_3_ perovskite layer, which involves first dip‐coating the substrate in PbI_2_, followed by dipping it into a CH_3_NH_3_I solution [24, 25].

Despite its advantages, solution coating faces significant hurdles requiring resolution. These include precise control of layer thickness and conformal coverage on non‐planar surfaces, minimization of precursor solution waste, and ensuring mechanical integrity.

#### 2.2.2 Vapor‐Assisted Deposition

Vapor deposition methods play a crucial role in the preparation of semiconductor fibers due to their ability to precisely control the chemical composition, structure, and thickness of thin films. This method is broadly categorized into physical vapor deposition and chemical vapor deposition.

Physical vapor deposition (PVD) primarily utilizes physical processes such as evaporation or sputtering to convert solid source material into a vapor phase, which then condenses onto a fiber substrate to form a thin film. This method is typically conducted in a vacuum or low‐pressure environment, relies mainly on physical processes, and generally does not involve chemical reactions of the deposited material itself. Its fundamental principle involves two key stages: (1) The solid source material is transformed into vapor‐phase atoms, molecules, or ions via physical means. (2) The vapor species transport through the vacuum or low‐pressure environment and impinge upon a cooler fiber substrate surface. They then form a solid thin film through processes of physical adsorption, condensation, nucleation, and growth. The chemical composition of the resulting film is usually identical to that of the source material. Its most common application is the deposition of electrodes within fiber devices, such as metals like gold and platinum.

Chemical vapor deposition (CVD) utilizes gaseous precursors that undergo chemical reactions on the substrate surface to generate solid thin film deposits, with gaseous byproducts being carried away. It is a mainstream technique for preparing high‐quality, large‐area semiconductor devices. This method goes beyond mere physical deposition by introducing chemical reactions either during or after the deposition process. Its basic procedure typically involves: (1) Gaseous precursors are transported to the vicinity of the fiber substrate. (2) Precursors undergo chemical reactions either on the substrate surface or in the gas phase near it, generating solid reaction products and volatile gaseous byproducts. (3) The solid products formed by the reaction deposit onto the fiber substrate surface, forming a thin film. Subsequent thermal treatment is often required to promote film crystallization, improve crystal quality, or adjust stoichiometry. Building upon CVD technology, methods have been developed for the in situ preparation of continuous semiconductor optoelectronic fibers directly on fiber substrates. For example, this approach employs the directional pyrolysis of silane (SiH_4_) precursors to in situ synthesize high‐purity, large‐grain polycrystalline silicon layers on the fiber substrate. This imparts both excellent electrical properties and low‐loss optical characteristics. Extensive existing research confirms CVD is an effective pathway for the efficient fabrication of such continuous semiconductor optoelectronic fibers [26].

Despite significant advancements, vapor‐assisted deposition still faces persistent challenges in uniform film deposition, precise compositional control, and overcoming substrate thermal stability limitations on geometrically complex 3D surfaces, which impede their scalability and functional integration.

#### 2.2.3 Thermal Drawing

Thermal drawing process is a core technology for continuous fiber fabrication achieved through high‐temperature melting, rheological control, and stretch‐forming, particularly well‐suited for manufacturing multifunctional composite fibers. Based on the intrinsic material transformations during processing, this process can be classified into two categories:

Physical thermal drawing: This type involves only physical state transitions of the materials being drawn, with no alteration of chemical composition or formation of new phases. Its core stages include: (1) Component materials within the preform melt into viscous fluids under high temperature. (2) Precise control of the temperature field and drawing rate enables matching and coordinated deformation of the melt's rheological behavior. (3) Upon cooling, a composite fiber with pre‐designed micro/nano structures is formed, with all components retaining their original chemical composition. A typical example is the fabrication of ultra‐flexible glassy semiconductor fibers. In this approach, a polyetherimide (PEI) polymer cladding with a softening temperature of 217°C mechanically supports and confines a (Te_85_Se_15_)_45_As_30_Cu_25_ glass core. At the drawing temperature, the viscosity of the PEI cladding is substantially higher than that of the telluride glass, allowing the polymer to bear the structural load and enabling the continuous production of thermoelectric fibers over hundreds of metres. Notably, this process relies solely on physical state transitions of both the polymer and the semiconductor core, without involving chemical reactions or compositional reconstruction [27].

Reactive thermal drawing accompanied by chemical changes: This type introduces chemical reactions during thermal drawing, generating new functional phases via in situ synthesis. Its key stages comprise: (1) The preform contains reactive precursor materials. (2) During the dynamic high‐temperature drawing process, thermally activated diffusion and reactions between precursors are triggered. (3) Reaction products crystallize into phases upon fiber cooling, forming functional composite materials synthesized in situ. Extending synthesis across the entire fiber length utilizing the high temperatures of the drawing process has been demonstrated. For instance, using aluminum (Al) as the reactive material, silicon (Si) fibers are obtained via thermal drawing; the aluminum melts into a liquid and reacts with a silica cladding, reducing the silica to silicon [28].

Thermal drawing exhibits significant potential for fabricating functional composite fibers, particularly semiconductor fibers. However, its successful implementation faces a series of formidable challenges. These challenges include, on one hand, overcoming processing difficulties arising from the semiconductor material's inherent high melting point, high‐temperature instability, and severe rheological mismatch with the cladding material. On the other hand, it necessitates the precise control of the physicochemical states at the interface between heterogeneous materials during the extreme high‐temperature, high‐speed stretching process to avoid detrimental reactions, interdiffusion, and stress‐induced damage, thereby preserving the superior performance of the semiconductor core and the integrity of the interfacial structure.

### 2.3 Operating Principles of Fiber Devices

A solar cell converts sunlight directly into electricity via the photovoltaic effect. Among emerging materials, perovskites have emerged as leading candidates for high‐performance fiber solar cells owing to their solution processability and exceptional defect tolerance, as reflected in most reported top‐performing devices. Fiber‐shaped perovskite solar cells (PSC) are adapted from planar PSC architectures, with several configurations explored to date. A representative design adopts a metal‐wire/electron transport materials (ETM)/perovskite/hole transport materials (HTM)/metal‐wire electrode stack, in which incident light absorbed by the perovskite layer generates electron–hole pairs that are subsequently separated and collected at the respective electrodes.

A photodetector converts incident photons into an electrical signal by generating and separating photo‐induced charge carriers. In photodetectors (PDs), light absorbed in the active layer creates carriers that are separated and transported under an external bias and/or a built‐in electric field to produce a photocurrent, thus transducing optical input into an electrical output. Based on structure and operation, PDs are broadly categorized as photoconductors, photodiodes, or phototransistors. Photoconductors, the simplest class, employ two electrodes on a semiconductor and require an applied voltage to separate carriers; typical devices consist of a photosensitive semiconductor channel bridged by two electrodes. Photodiodes, commonly implemented as p‐n, p‐i‐n, or Schottky junctions, feature a built‐in barrier that enables low dark current and efficient carrier separation even at low or zero bias. Phototransistors incorporate source, drain, and gate electrodes separated by a dielectric, allowing gate‐controlled charge transport.

An LED performs the reverse process, converting electrical energy into light through radiative recombination of electrons and holes within a semiconductor. In LED fibers, an emissive layer is sandwiched between the HTM and ETM to form a double heterojunction structure. Under an applied bias, carriers are injected from the electrodes through the transport layers into the perovskite emitter, where they recombine radiatively to generate photons.

## 3 Junction‐Free Architectures in Semiconductor Fibers

### 3.1 Semiconductor in‐Fiber Deposition

**FIGURE 3:**
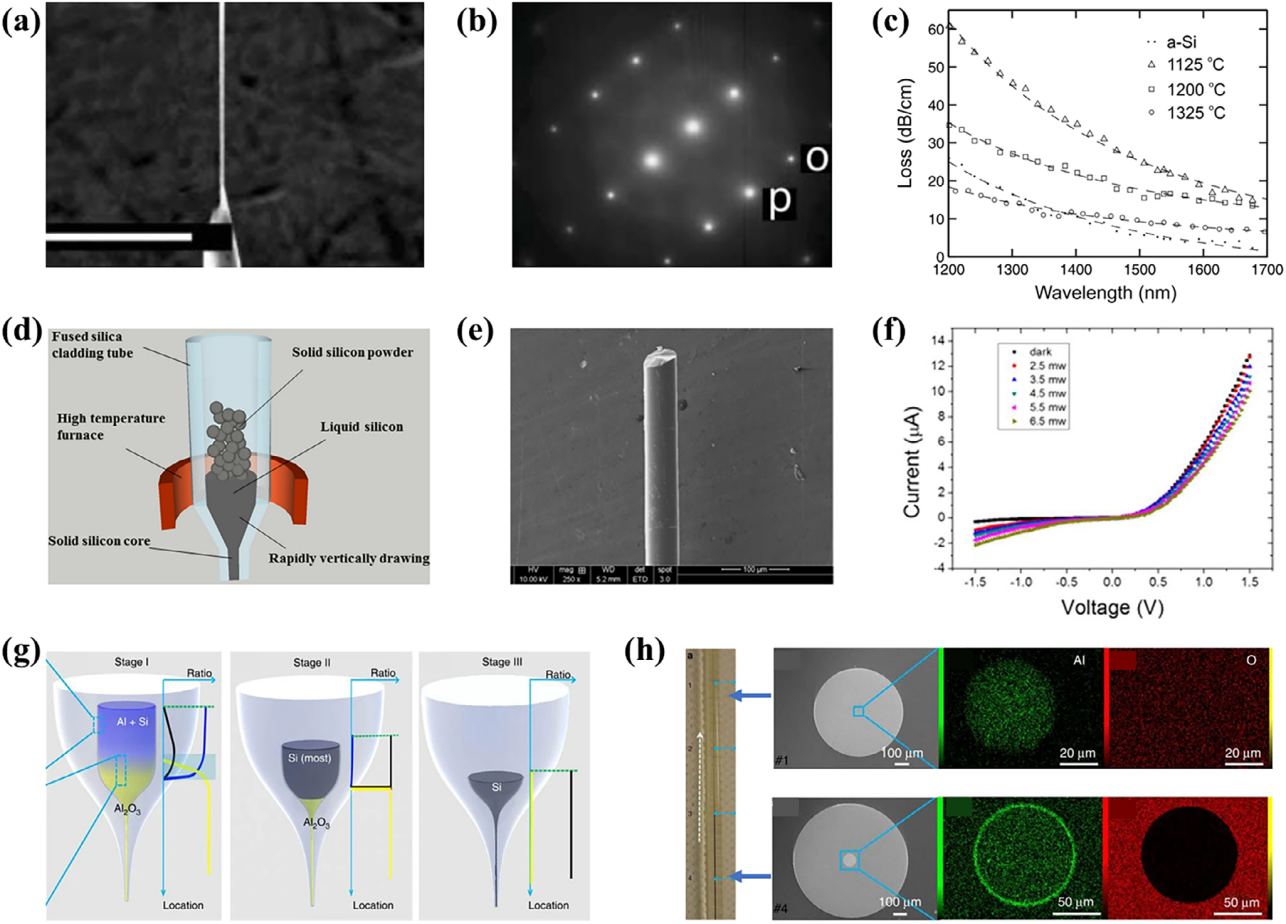
(a) SEM micrograph of an 80‐µm‐diameter section of a single‐crystal silicon wire. Scale bar, 50 µm. (b) Electron diffraction pattern collected from a cross‐sectional slice of the single‐crystal wire. Reproduced with permission [29] Copyright 2006, The American Association for the Advancement of Science. (c) Transmission losses as a function of wavelength for amorphous sample and polysilicon samples. Reproduced with permission [26] Copyright 2010, AIP Publishing. (d) A schematic illustration showing the vertical drawing fabrication process for making a Si‐cored fiber. (e) An SEM image of a Si fiber without silica cladding. (f) *I*–*V* characteristics of a Schottky photodetector. Reproduced with permission [30]. Copyright 2015, AIP Publishing. (g) Sketches of the evolution of the core in the heating zone throughout the drawing process. (h) The optical image, SEM images, and EDS mapping of a piece of fiber where a light‐yellow core transitions to a black core. Reproduced with permission [28] Copyright 2015, Springer Nature.

Elemental semiconductor fiber cores provide the foundation for fiber‐based optoelectronic devices. These versatile structures can function directly as individual elements within devices, be bundled into flexible two‐dimensional （2D ） arrays, or assembled into complex architectures exhibiting anisotropic behavior and spatial resolution. The field emerged in 2006 with the first reports of fibers featuring elemental semiconductor cores, initially prepared via chemical reactions confined within prefabricated fiber templates. Sazio, P. J. et al. pioneered the first semiconductor‐core fiber by fabricating a silicon core within glass capillaries using high‐pressure chemical vapor deposition (HPCVD). This method employed pressurized SiH_4_ precursor gas to deposit silicon inside the hollow channels. As a result, a single‐crystal silicon wire with a diameter of 1.6 mm, growing in the <112> direction, was obtained (Figure 3a,b). Crucially, the distinct, independent deposition steps inherent to CVD enable considerable design flexibility, facilitating the subsequent integration of other functional materials, such as germanium for optoelectronic functionality or metals, directly into fibers [29].

Silicon is typically deposited at low temperatures in an amorphous state. This amorphous silicon can subsequently be crystallized into polysilicon via post‐deposition high‐temperature annealing. Through optimization of both the deposition and annealing processes, high‐quality amorphous silicon and polysilicon core materials with low optical losses have been achieved (Figure 3c) [26].

Soon afterward, a more conventional thermal drawing method was demonstrated, which has since been widely adopted by the community. Advanced demonstrations of incorporating silicon into fibers for electronic and photonic applications have been achieved via a physical flow‐based thermal drawing process, in which high‐purity silicon is embedded in a silica preform and drawn into fibers with a molten silicon core. Such a rapid vertical drawing method allowed the production of meter‐long circular silicon fiber cores in a few minutes. As shown schematically in Figure 3d, the process involves packing silicon powder into a fused silica tube, heating the assembly to melt the silicon and soften the silica, and then vertically drawing it into fiber form. Analysis confirmed that the silicon core was continuous (Figure 3e) [30, 31].

Such conventional thermal drawing methods have been adapted to incorporate a range of materials within the core, and germanium and compound semiconductor fibers have been fabricated for use in applications ranging from sensing to electro‐optic modulation, thermoelectric conversion, and photovoltaic conversion. For example, inorganic micro/nanowires have been seamlessly integrated in a fiber‐like carrier using thermal drawing technology. The resulting p‐type Bi_0.5_Sb_1.5_Te_3_ and n‐type Bi_2_Se_3_ fibers are intrinsically crystalline, highly flexible, ultralong, and mechanically stable, while maintaining high thermoelectric properties as their bulk counterparts [32]. While conventional inorganic semiconductor fibers require rigid glass cladding due to high material melting points, Zhang et al. achieved ultra‐flexibility by co‐drawing a (Te_85_Se_15_)_45_As_30_Cu_25_ semiconducting glass core with a polyetherimide (PEI) polymer cladding. The resulting fiber sensor operates across wide temperature ranges with high sensitivity/accuracy and sustains bending radii < 2.5 mm. Demonstrated in a woven 3 × 3 textile array, it simultaneously maps temperature distribution and locates heat/cold sources with millimeter spatial resolution [27].

Significantly, the silica glass cladding can also serve as a microcrucible for modifying the core material, either during or after fabrication. For example, reactive formation of silicon fibers from an aluminum core has been reported. At the high drawing temperature (≈2200°C), Al reduces SiO_2_ to silicon (Si), producing Al_2_O_3_.

$$4Al+3SiO_{2}\to 2Al_{2}O_{3}+3Si$$

Density‐driven phase separation occurs concurrently, forming a pure Si core within the silica fiber. The process comprises three stages (Figure 3g): (1) Reaction initiation and Al_2_O_3_ separation, (2) Completion of redox consumption, and (3) Si core fiber formation. Figure 3h confirms the core transition via optical/EDS mapping. This reactive drawing technique significantly enhances production scalability and enables complex core geometries. It demonstrates the feasibility of simultaneous chemical synthesis and fiber fabrication, expanding material and architectural possibilities for fiber devices [28].

### 3.2 Semiconductor Morphological Regulation

Thermal drawing enables geometrically faithful miniaturization, transforming macroscopic preforms into micro‐scale fibers while preserving cross‐sectional ratios. Therefore, this technology holds great potential for the production of continuous semiconductor features at the nanoscale, including continuous semiconductor wires or directly fabricated nanowire arrays with very high aspect ratios. Through a single draw, the draw‐down ratio is physically limited to the range of a few tens to one hundred. Also, a single‐core design is commonly adopted due to the difficulty of drilling multiple holes in the cladding material to accommodate many cores.

**FIGURE 4:**
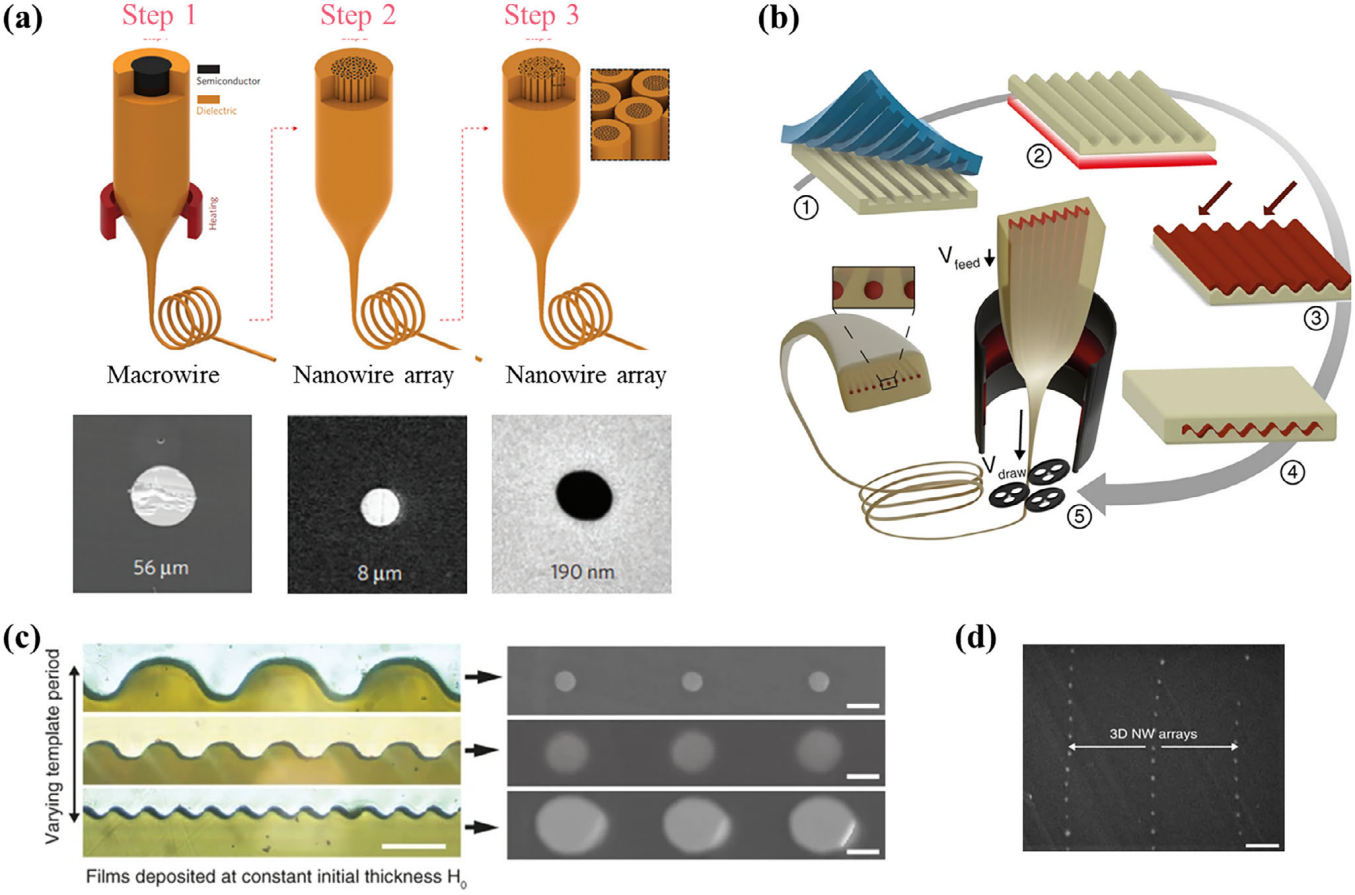
(a) A macroscopic multi‐material rod is reduced to ordered arrays of nanowires by thermal size reduction in successive steps. Reproduced with permission [33]. Copyright 2011, Springer Nature. (b) A single‐step drawing process that employs a templated fiber preform combined with glancing‐angle deposition. (c,d) Images of the films after deposition onto a polymer substrate with different template periods and the corresponding nanowire array geometries. Reproduced with permission [35]. Copyright 2011, Springer Nature.

One method to manufacture ordered nanoarrays is iterative co‐drawing based on the stack‐and‐draw technique (see Figure 4a), whereby a single‐core fiber is drawn, cut into equal sections, assembled into a bundle, and re‐drawn. Reaching the nanoscale typically requires three successive draws. The draw‐down ratio increases exponentially through an iterative stack‐and‐draw process, resulting in a significant reduction in fiber size. For example, macroscopic rods are reduced by 25‐ to 300‐fold at each step. Using a reduction factor of 50–100 for three iterative steps, a 10 mm initial rod is reduced to hierarchically ordered structures of around 10 nm. As a result, globally oriented, endlessly parallel, axially and radially uniform semiconducting nanowire arrays that are hundreds of metres long, with nanowire diameters less than 15 nm, are obtained as well‐ordered high‐density arrays. Such a method has been proven to be applicable to a variety of low‐melting‐temperature metals, metal alloys, and polymers, such as the glass‐forming chalcogenide Ge_15_As_25_Se_15_Te_45_, metallic Sn, SnPb alloys, SnAg alloys, hollow‐core polyvinylidene fluoride (PVDF) tubes, and As_2_Se_3_‐PVDF core‐shell nanowires. Finally, the size‐dependent photoconductivity of selenium micro‐ and nanowire arrays has been demonstrated. Photoconductance scales logarithmically with reduced nanowire dimensions and increased array density. The I/I0 gain rises by one order of magnitude for step‐2 microwires and by two orders of magnitude for step‐3 nanowires versus the initial single microwire. Smaller nanowires exhibit accelerated on/off switching [33]. Zhang et al. used this stack‐and‐draw technique to draw tin selenide (SnSe) fibers, thereby further reducing the semiconductor fiber size while increasing the number of cores. Double‐, triple‐, and quadruple‐core SnSe fibers were fabricated via this method for a 2D thermoelectric fabric [34].

However, the regularity of fiber spacing and filament diameter is limited by the precise stacking and uniformity of the previous steps. William Esposito et al. overcame this limitation by developing a single‐step drawing process for large‐scale, meter‐long ordered arrays (Figure 4b). Their method employs a templated fiber preform combined with glancing angle deposition to deliberately induce film thickness fluctuations. After drawing, this yields highly ordered, polymer‐encapsulated chalcogenide glass nanowires with diameters down to 50 nm (Figure 4c). Crucially, the technique enables independent control over nanowire diameter and spacing, providing unprecedented transverse geometrical tuning. Furthermore, stacking multiple templated films within a single preform allows fabrication of 3D nanowire assemblies (Figure 4d), facilitating integrated multifunctional photonics at reduced scales [35].

### 3.3 Semiconductor Crystallization Regulation

**FIGURE 5:**
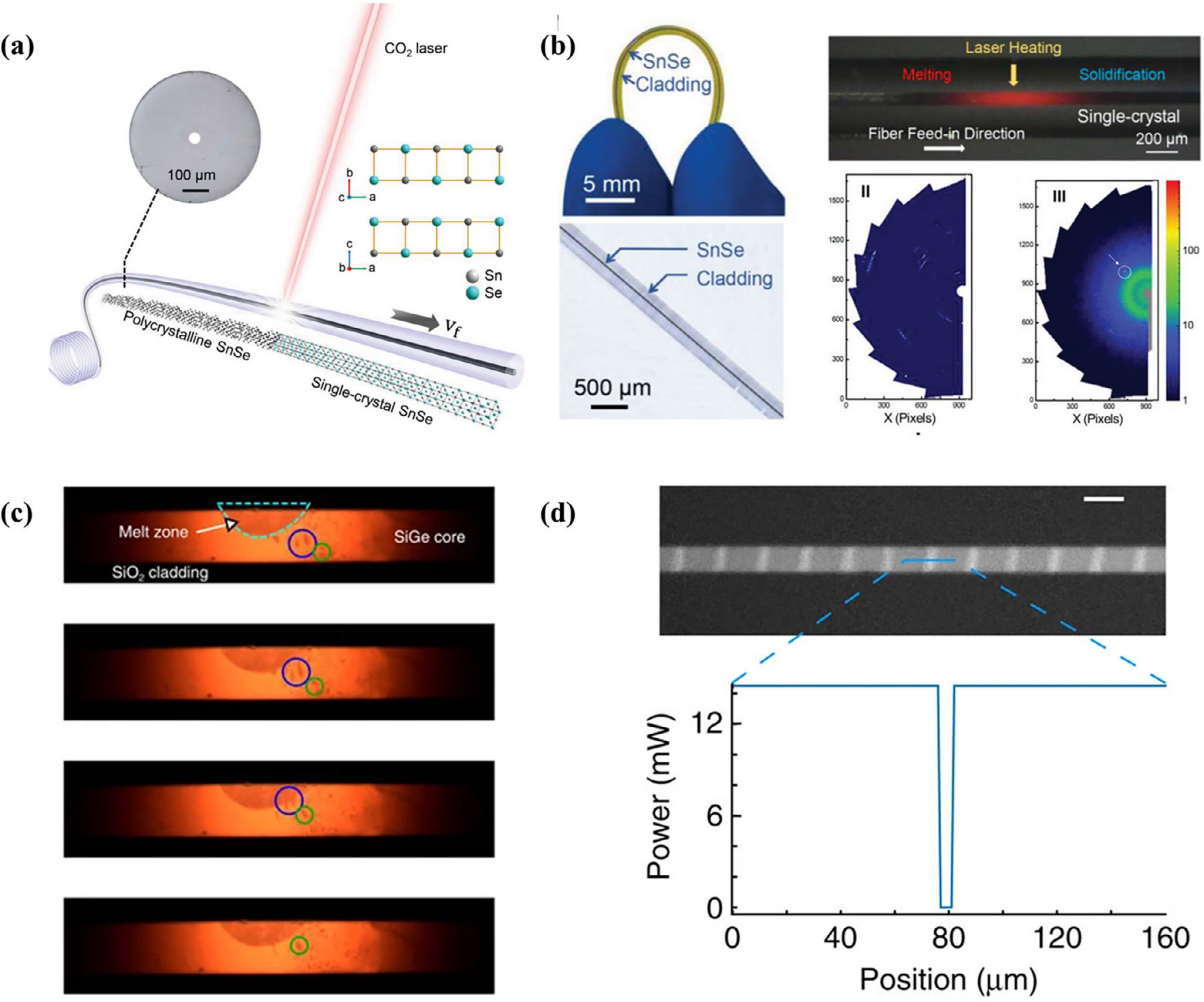
(a) Schematic illustration and simulated temperature profile of the post‐draw laser recrystallization process for a single SnSe fiber. (b) Schematic illustration of Ge‐rich liquid flowing from the untreated region to the laser induced melt zone. Reproduced with permission [14], Copyright 2020, WILEY‐VCH Verlag GmbH & Co. KGaA, Weinheim. (c) Ge‐rich grating formed in the fiber core by periodically interrupting the laser beam. (d) Power versus distance profile for the germanium grating process. The angle of the grating is due to asymmetric heating and the resulting tilted solidification boundary. Reproduced with permission. Reproduced with permission [36], Copyright 2016, David A. Coucheron et al.

Thermal drawing imposes high processing rates and therefore far from equilibrium conditions on semiconductor crystallization. Rapid quenching during fiber cooling triggers heterogeneous nucleation, yielding polycrystalline or amorphous structures. Grain boundaries in these polycrystalline fibers act as scattering centers for electrons and photons, segregate impurities, and introduce mechanical defects. Critically, defect‐rich boundaries and poorly crystallized regions promote carrier recombination, limiting device performance. These inherent limitations underscore the challenge of directly producing high crystallinity semiconductors, or even single crystalline semiconductor fibers, via thermal drawing. Although it is rather difficult to control the quality of crystals during the heating process, the laser heated pedestal growth method has enabled faster and more effective preparation than other crystal growth methods, achieving high quality single crystal fibers with uniform diameters and specific crystallographic directions. However, for thermally drawn fibers, efforts to produce single crystal fibers have mainly focused on achieving superior optical properties. In this context, Zhang et al. developed a laser recrystallization technique for thermally drawn fibers. As illustrated in Figure 5a, a focused CO_2_ laser is employed (with a 500 µm spot) to scan SnSe fibers, creating a stable melt zone controlled by power and scan rate. Precise thermal gradient management promotes single‐crystal formation along the entire fiber length, verified by multi‐position X‐ray diffraction (XRD) and wide angle X‐ray scattering (WAXS). Figure 5b demonstrates woven single crystalline SnSe fibers in textiles harvesting body heat for electricity generation [34].

In addition to enhancing crystallinity, another advantage of laser induced recrystallization is the ability to adjust the alloy composition, alleviate component segregation, and homogenize the band gap and optical properties of alloy semiconductor materials within the fibers. For example, SiGe alloy cores form single‐phase solid solutions crystallizing between 938°C and 1414°C, with bandgap and refractive index tunable via Si:Ge ratio. During finite rate solidification, kinetic segregation creates silicon‐rich precipitates and compositional gradients due to limited solid state diffusion (Figure 5c). Utilizing CO_2_ laser irradiation through the glass cladding enables core recrystallization, enhancing optical transmission. This approach suppresses constitutional undercooling at high solidification velocities while validating microscale solidification models. Precise control of recrystallization conditions permits either extended uniform single crystals or intentional compositional microstructures (Figure 5d) within the fiber core [36].

## 4 Axially Continuous Radial Heterojunction Fiber Architectures

The combination of conductors, semiconductors, and insulators with well‐defined geometries and at prescribed length scales while forming intimate interfaces is essential for most functional electronic and optoelectronic devices. In planar devices, these structures are typically produced using a variety of elaborate wafer‐based processes, which allow for small features but are restricted to planar geometries and limited coverage areas. For semiconductor materials and electrode materials that can be processed by solution methods, such as perovskite materials and carbon electrode materials, large scale production can be achieved through means like solution spraying and roll‐to‐roll printing. However, creating functional devices on non‐planar substrates, such as cylindrical optical fibers, presents distinct challenges. Conventional planar fabrication techniques are inherently limited in conforming to such geometries and achieving extensive coverage along the fiber length. Against this background, strategies to functionalize optical fibers have emerged. Most common inorganic semiconductor fibers adopt radial heterojunction architectures, which include coaxial core–shell and laterally aligned parallel configurations. Such architectures can be formed either by integrating metals with semiconductors or by combining different semiconductor materials.

### 4.1 Metal/Semiconductor Heterojunctions

To functionalize optical fibers, a first strategy relies on wafer‐based techniques and modified deposition to integrate functional materials at the tip or within a few tens of centimeters of microstructured silica fibers. As noted in Section 1, advanced HPCVD addresses key challenges in precursor decomposition, crystallization, and interface control, enabling the deposition of tailored materials directly within fiber pores. This chemical approach offers flexible control over material composition, crystallinity, and layer thickness. For instance, using group‐IV hydride precursors, amorphous germanium layers can be deposited at low temperatures and can completely fill the pore. Subsequent annealing at elevated temperatures converts these amorphous layers into polycrystalline semiconductors. In this way, HPCVD provides a pathway to integrate active optoelectronic functionalities within fiber platforms, effectively merging semiconductor device physics with microstructured optics.

**FIGURE 6:**
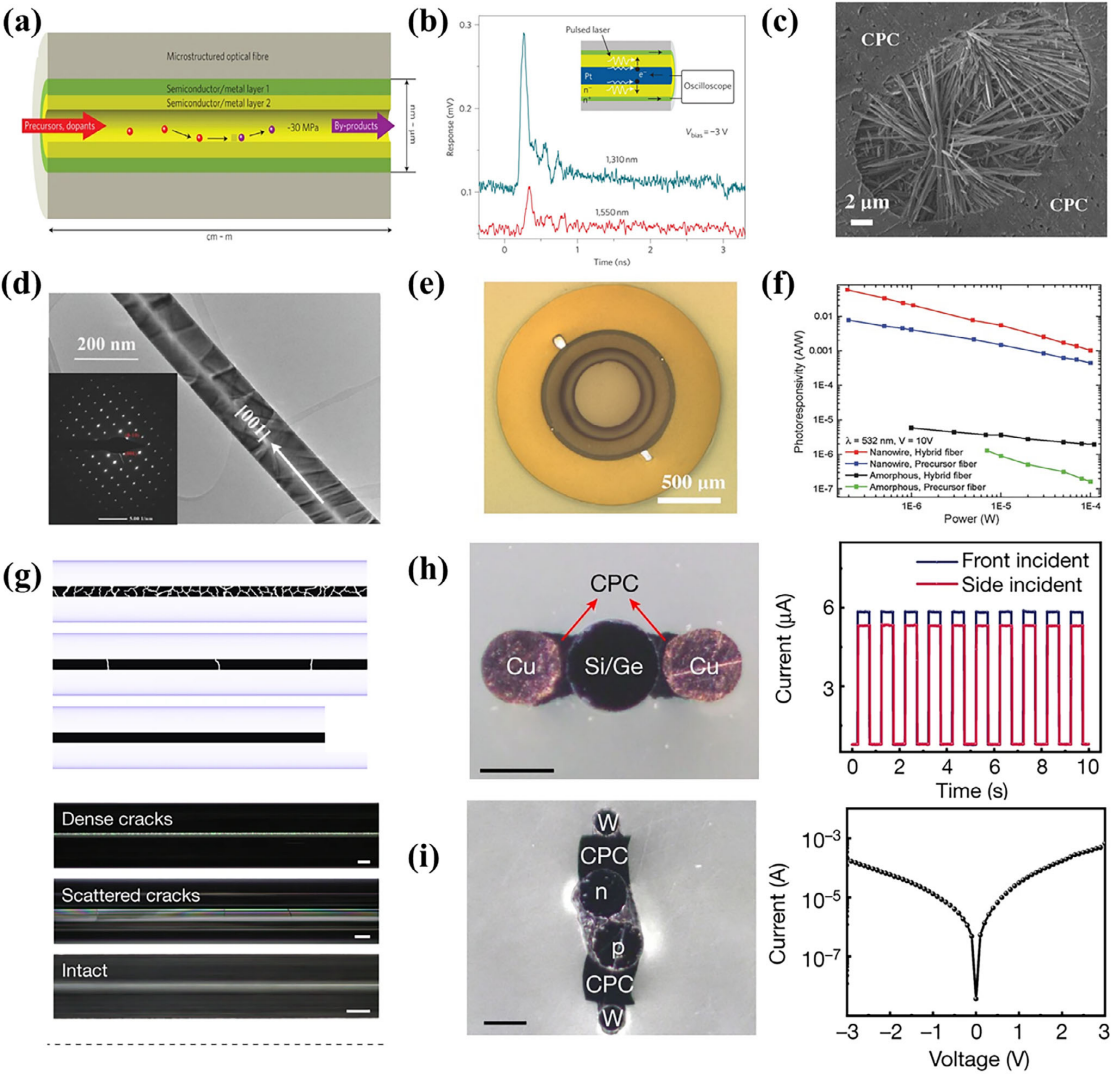
(a) Illustration of HPCVD in the microstructured optical fibers. (b) Photodetection response of a Pt‐Si diode to ∼10 ps laser pulses at wavelengths of 1310 and 1550 nm. Reproduced with permission [37]. Copyright 2012, Springer Nature. (c) Scanning electron microscopy (SEM) image of Se nanowires grown between the CPC contacts. (d) Bright‐field transmission electron microscopy (TEM) image and selected area electron diffraction (SAED) pattern (inset). (e) Optical photograph of the cross section of the fiber. (f) Comparison of photoresponsivity of amorphous‐based and nanowire‐based fibers. Reproduced with permission [40]. Copyright 2017, WILEY‐VCH Verlag GmbH & Co. KGaA, Weinheim. (g) Fiber core geometries corresponding to different stress levels. Schematics and optical images of fibers show intact and cracked cores in response to the stress formed during fabrication. Scale bars, 100 µm. (h) Optical images of the side‐view and cross‐section of the single‐core optoelectronic fiber, where the core semiconductor is connected to each copper electrode through a layer of CPC. The resulting optoelectronic fiber shows a pseudo‐omnidirectional response that maintains sensitivity for different directions. Scale bars, 50 µm. (i) Optical images of the side‐view and cross‐section of the dual‐core p‐n junction fiber. The *I*–*V* characteristic of the p‐n junction fiber. Scale bars, 50 µm. Reproduced with permission [41]. Copyright 2024, Zhixun Wang et al.

Building on this approach, He et al. demonstrated the integration of precisely doped semiconductor materials and high quality rectifying junctions directly into microstructured optical fibers, enabling high speed, in‐fiber functionalities such as photodetection at telecommunications wavelengths. Sequential deposition in Figure 6a allows for the formation of complex, multilayer structures including a platinum/n‐type silicon (Pt/n‐Si) heterojunction within 15 µm diameter channels. The resulting heterojunction exhibits a Schottky barrier height of approximately 0.8 eV, making it suitable for high speed photodetection at 1300–1550 nm (Figure 6b). The fabricated Pt/n‐Si heterojunction shows excellent diode characteristics with a low dark current of 0.27 pA µm^−2^ at −1 V and a responsivity of 1.4 mA W^−1^ at −3 V, with performance comparable to planar NiSi/n‐Si devices [37].

An alternative approach exploits the well‐established thermal drawing of macroscopic preforms that integrate the desired multi‐material architecture. Researchers have demonstrated scalable thermally drawn functional fiber photodetectors with amorphous semiconductor cores contacted by metallic microwires. Preforms comprising low‐melting‐temperature conductors, semiconductor cores, and polymeric insulators are drawn into extended fiber lengths, achieving intimate metal–semiconductor interfaces with sub‐100 nm features. This technique transforms geometrically defined but non‐functional preforms into operational devices through interfacial consolidation during drawing. The resulting fibers exhibit photoconductive behavior across their entire length, effectively forming a one‐dimensional (1D) photo‐detecting element. Notably, these fibers enable novel large‐area sensing architectures. Woven fabrics incorporating such fibers can localize illumination points on a surface using only order‐*N* detection elements, significantly more efficient than conventional 2D arrays requiring *N* ^2^ elements. Stacking fabric layers further permits directional sensing of incident light [38].

To enhance the performance of fiber‐based photodetectors, it has been established that the geometric parameters of cylindrical architectures play a critical role in determining their sensitivity. Sorin et al. conducted a comparative study of the responsivity, noise characteristics, and sensitivity of fiber‐based photodetectors as a function of structural design and geometric scaling parameters. It was demonstrated that a reduction in fiber diameter correlates linearly with enhanced sensitivity, establishing a fundamental design principle for optimizing photodetector performance. Precise control over the submicron‐scale dimensions affords more than an order of magnitude increase in the fiber‐device sensitivity [39].

Another powerful strategy for microstructural regulation and optoelectronic performance enhancement involves the growth of well‐oriented semiconducting nanowires. By combining thermal drawing of amorphous selenium domains with subsequent sonochemical nanowire growth, crystallization is guided along the [001] direction, dictated by the intrinsic anisotropy of trigonal‐phase selenium (Figure 6c,d). Crucially, the polymer fiber platform allows precise and arbitrary positioning of active devices within the fiber cross‐section. This capability enables the realization of a fluorescence‐imaging fiber in which two nanowire devices arranged circumferentially around a step‐index core operate simultaneously as light sources and photodetectors with enhanced responsivity (Figure 6e,f) [40].

In addition, the fabrication of ultra‐long, continuous, fracture‐free semiconductor fibers remains fundamentally challenged by core fracture propagation, which originates from complex interfacial stresses developed between the glass cladding and semiconductor core during processing. Wang et al. revealed that the stress development and mechanical behavior during the three critical stages of thermal drawing, including viscous flow, molten core crystallization, and cooling, can be systematically analyzed. Based on the viscoelastic and viscous properties of glass materials, quartz glass and aluminosilicate glass were selected as claddings for silicon and germanium core materials, respectively. This selection minimized radial stress at the core‐cladding interface, enabling the preparation of high‐quality continuous semiconductor fibers. The work provides theoretical guidance for high‐temperature thermal drawing fiber technology, emphasizing two key principles: (1) matching the cladding glass annealing point to the core material melting point, and (2) aligning the cladding thermal expansion coefficient with that of the core material.

Following the preparation of continuous long fibers, the glass cladding was removed via HF etching, yielding a standalone semiconductor fiber. This fiber was then integrated with metal wires, a conducting polymer, and an insulating polymer into a single composite structure through convergent fiber drawing. Intimate interfaces between components were formed within the necking region (Figure 6h). Leveraging an in‐fiber self‐assembly strategy, optoelectronic fibers based on silicon and germanium have been fabricated with precisely defined rectangular cross‐sections (300 × 200 µm) and integrated back‐to‐back Schottky contacts. As prepared, Si‐based fibers exhibit pseudo‐omnidirectional under 532‐nm illumination at a 2 V bias, with measurable sensitivity to both front and side incidence (Figure 6h). Furthermore, self‐assembled p‐n junction fibers demonstrate clear rectifying behavior, as confirmed by their *I*–*V* characteristics (Figure 6i) [41].

### 4.2 Semiconductor p‐n Junction

Flexible silicon p‐i‐n junction fibers, fabricated via high‐pressure chemical vapor deposition (HPCVD), present significant opportunities for textile photovoltaics and optoelectronics. These fibers exhibit photovoltaic functionality and photodetection capabilities. As demonstrated by He et al., the HPCVD process produces crystalline silicon p‐i‐n photodiode junction fibers offering superior quantum efficiency compared to earlier HPCVD‐fabricated metal‐silicon Schottky junction fibers. This crystalline Si structure enables high‐performance optoelectronic functionality unattainable with organic or non‐crystalline materials. Specifically, the fibers function as high‐speed photodetectors with 1.8 GHz bandwidth. They achieve photodetection responsivities of ≈0.3 A W^−1^ at 633 nm under zero/negative bias, significantly exceeding those of Pt‐Si Schottky fiber detectors. This enhanced performance stems from the large built‐in electric field within the i‐layer, enabling rapid carrier sweep out.

As photovoltaic devices under AM 1.5 illumination, the fibers generate rectifying *I*–*V* characteristics, yielding a *V*_OC_ of 0.22 V, *J*_SC_ of 2 mA cm^−2^, FF of 0.55, and 0.5% efficiency [42]. The efficiency of silicon‐based fiber solar cells is primarily limited by suboptimal layer thicknesses, reducing light absorption, and by low crystalline quality, which induces intolerable defects. Despite widespread research, their inherently low performance has constrained field development [42, 43, 44].

**FIGURE 7:**
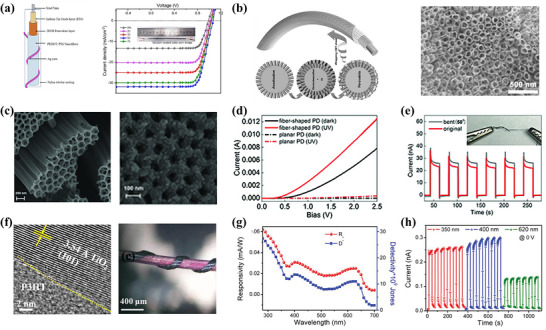
(a) Schematic illustration of the fiber‐shaped solar yarn architecture and the *J*–*V* curves. Reproduced with permission [23]. Copyright 2022, Donghua University, Shanghai, China. (b) Schematic illustration of the fabrication and morphology of TiO_2_ nanotubes. Reproduced with permission [47]. Copyright 2016, WILEY‐VCH Verlag GmbH & Co. KGaA, Weinheim. (c) Morphology of p‐CuZnS/n‐TiO_2_ heterojunction. (d) *I*–*V* characteristics of the fiber‐shaped UV photodetector in the dark and under UV illumination. (e) On/off switching tests of the fiber‐shaped photodetector at the virgin state and the bent state (≈ 50°). Reproduced with permission [48]. Copyright 2018, WILEY‐VCH Verlag GmbH & Co. KGaA, Weinheim. (f) High‐resolution transmission electron microscopy (HRTEM) images of TiO_2_/P3HT heterojunction and an optical photograph of the device. (g) Responsivity and detectivity as a function of wavelengths at zero bias. (h) *I*–*t* curves. Reproduced with permission [49]. Copyright 2020, WILEY‐VCH Verlag GmbH & Co. KGaA, Weinheim.

Solution‐processable perovskites enabled the first functional fiber‐shaped solar cells via dip coating. In 2014, Peng et al. pioneered this approach by sequentially depositing TiO_2_ slurry, MAPbI_3_ perovskite solution, and an OMeTAD hole‐transport layer, forming an in situ TiO_2_/perovskite/OMeTAD heterojunction directly on the curved stainless‐steel wire. As shown in Figure 7a, a carbon nanotube outer electrode was then assembled, and this solution‐coated device achieved a PCE of 3.3% (*V*_OC_ = 0.664 V, *J*_SC_ = 10.2 mA cm^−2^, FF = 0.487), establishing the viability of fiber‐based photovoltaics [45].

During solution coating, the composition of perovskite precursor solutions critically influences crystallization pathways and film quality on curved surfaces, ultimately determining the performance of fiber‐shaped devices. The fabrication of high‐quality perovskite layers via dip‐coating critically depends on precise control over ink viscosity and surface tension. To address this, a hybrid perovskite quantum dot ink system comprising poly(triarylamine) (PTAA), 1,3,5‐tri[(3‐pyridyl)‐phen‐3‐yl]benzene (TmPyPB), and perovskite QDs was engineered for the deposition of active layers in fiber devices. This formulation yields ultra‐smooth QD films. Consequently, monolithic fiber‐based devices achieve seamless bifunctional light‐emitting/photodetecting integration [46]. Chloride additives, known to facilitate crystallization within mesoscopic scaffolds, were used as catalysts for the preferential formation of vertically oriented 2D‐3D hybrid perovskite crystals. After chlorine incorporation, the hybrid structures exhibit enlarged grains, smooth morphology, and extended charge carrier lifetimes (Figure 7g). This optimization enables Sn‐based devices with an ITO/2,2’,7,7’‐Tetrakis‐(N,N‐di‐4‐methoxyphenylamino)‐9,9’‐spirobifluorene/2D‐3D perovskite/ Poly(3,4‐ethylenedioxythiophene) heterojunction structure to achieve a record PCE of 11.96% [23].

Vertically aligned TiO_2_ nanotubes grown via electrochemical anodization using a fluorine‐containing solution exhibit enhanced electron transport and enlarged specific surface area. Peng et al. applied this solution‐processed method to titanium wires, where nanotube diameters were precisely controlled by the anodization voltage (Figure 7b). Subsequent cathodic deposition of PbI_2_ precursors into these pores created a porous scaffold with superior CH_3_NH_3_I infiltration capacity compared with conventional dip‐coating, enabling complete perovskite conversion. This TiO_2_/TiO_2_‐nanotube/perovskite architecture achieved 7.1% PCE in fiber solar cells [47].

The cost‐effective anodization of titanium enables the fabrication of diverse TiO_2_ nanorods on Ti wire substrates. These 1D hybrid nanostructures enhance device performance by improving optical/electrical properties, including prolonged charge carrier lifetimes, superior charge separation, and increased carrier generation. Leveraging TiO_2_’s wide bandgap (∼3.2 eV), which is ideal for UV‐A/B detection, and the high specific surface area inherent to its nanostructured forms, researchers have developed UV detectors incorporating hybrid heterojunctions. The first real‐time wearable UV radiation sensor that reads out ambient UV power density and transmits data to smartphones via Wi‐Fi is demonstrated. A p‐CuZnS/n‐TiO_2_ UV photodetector with high performance is successfully developed (Figure 7c). The fiber‐shaped device shows an outstanding responsivity of 640 A W^−1^, external quantum efficiency of 2.3 × 10^5^%, and photocurrent of ≈4 mA at 3 V, exceeding those of most current UV photodetectors at that time (Figure 7d,e). Its ultrahigh photocurrent enables it to be easily integrated with commercial electronics to function as a real‐time monitoring system [48].

Similarly, a TiO_2_/poly(3‐hexylthiophene) (P3HT) inorganic‐organic heterojunction (Figure 7f) is easily prepared by a combination of anodization and vacuum dip‐coating methods. The device structure is displayed in Figure 7f, where carbon nanotube yarn is intertwined tightly around the fibrous sample to ensure intimate and stable contact. The photocurrent increases slightly under illumination of visible light (620 and 400 nm), while it increases dramatically under UV light, consistent with the responsivity spectra in Figure 7g. The optimal flexible fibrous photodetector yields an ≈700% responsivity enhancement at 0 V under 350 nm illumination. The sharp cut‐off edge and high UV–vis rejection ratio indicate a self‐powered flexible UV photodetector (Figure 7h) [49].

Achieving uniform active layer thickness is a prerequisite for high‐performance fiber devices, particularly in large‐area textile‐integrated systems. To address this challenge, Shi et al. developed a micro‐pinhole‐assisted coating strategy that precisely regulates the deposition of functional materials. In their approach, a conductive yarn was first dip‐coated with a ZnS phosphor slurry and subsequently passed through a custom‐designed micro‐pinhole. This structure acts as a mechanical regulator, enabling simultaneous circumferential uniformization, longitudinal smoothing, and precise control over coating thickness. The result is a continuous, uniform phosphor layer that is critical for consistent device performance. Utilizing this method, the authors fabricated a 6‐m‐long, 25‐cm‐wide electroluminescent display textile incorporating approximately 5 × 10^5^ uniformly spaced luminescent units at 800‐µm spacing [50].

## 5 Axially Segmented Radial Heterojunction Fiber Architectures

### 5.1 Metal/Semiconductor Heterojunctions

**FIGURE 8:**
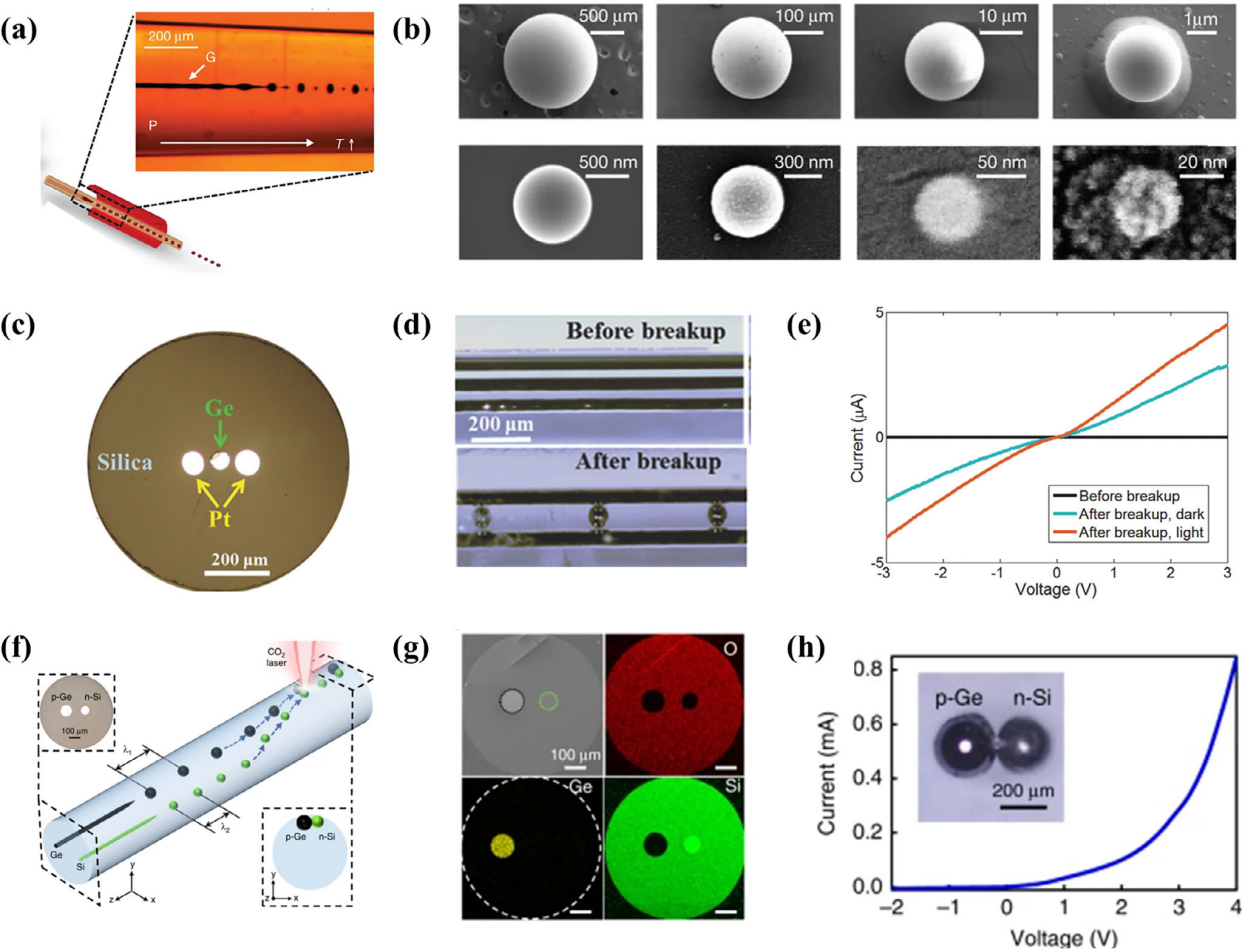
(a) Schematic illustration showing that a temperature gradient is applied along the axis to induce the formation of composite structured spherical particles. (b) SEM images of microparticles and nanoparticles. Reproduced with permission [51]. Copyright 2012, Springer Nature. (c) The silica‐cladding fiber contains one central semiconductor (germanium) core flanked by two metal (platinum) cores. (d) Side views of the triple‐core fiber before and after the selective breakup process. (e) Photoconductance of a triple‐core fiber before and after the selective breakup process in the dark and under the illumination of a 1550 nm laser. Reproduced with permission [53]. Copyright 2016, WILEY‐VCH Verlag GmbH & Co. KGaA, Weinheim. (f) Schematic of the asynchronous breakup of a p‐type germanium and n‐type silicon dual‐core fiber and the in‐fiber structure reconstruction based on CO_2_‐laser‐induced thermocapillary convection for fabricating p‐n heterojunction molecules. (g) The energy‐dispersive X‐ray spectroscopy (EDS) mapping of the polished cross‐section of the p‐type germanium and n‐type silicon dual‐core fiber. (h) *I*–*V* characteristic of the dual‐sphere p‐n germanium‐silicon heterojunction molecule. The inset shows the contacted p‐Ge/n‐Si junction molecule after processing. Reproduced with permission [54] Copyright 2019, Jing Zhang et al.

Joshua J. Kaufman et al. demonstrated that harnessing the Plateau‐Rayleigh capillary instability inherent in fiber drawing offers a scalable nanofabrication route. Their approach leverages the intrinsic scalability of fiber production to fabricate uniformly sized, structured spherical particles across an exceptional size range from 2 mm down to 20 nm (Figure 8a,b). Within this process, the fiber core and cladding act as the dispersed and continuous phases, respectively, which are solidified in situ upon cooling. By pre‐arranging diverse structures and materials within the macroscopic fiber preform, composite structured spherical particles can be directly produced [51].

Crucially, volume conservation dictates that spheres formed from core breakup possess diameters larger than their precursor cylinders. Consequently, spheres detaching from closely spaced cores can contact each other, forming multi‐sphere clusters or establishing electrical connections with integrated electrodes (contact‐by‐breakup), thereby enabling optoelectronic devices and complex junctions. For example, a fiber with dual cores, p‐type and n‐type silicon, is drawn and processed into spheres. Spatially coherent breakup leads to the joining of the spheres into a bispherical silicon p‐n molecule. The resulting device exhibits a rectifying *I*–*V* curve consistent with the formation of a p‐n junction [52].

The significant difference in material melting points enabled selective breakup via fluid instability, targeting only the semiconducting core while leaving the metal cores continuous. Thus, a preform composed of fused silica cladding and three distinct cores (a central germanium rod flanked by two platinum rods, see Figure 8c) was utilized. This selective process fragmented the germanium core into a series of relatively larger spheres, establishing direct contact between each sphere and the two continuous platinum filaments, resulting in an integrated ladder‐type structure within the silica fiber (Figure 8d). This structure exhibited measurable photosensitivity. The realization of direct in‐fiber electrical contact between crystalline semiconductor spheres and metal electrodes was confirmed by applying a bias voltage across the Pt electrodes. Prior to selective breakup, negligible current response indicated an open circuit (Figure 8e). Following breakup, measurable current was observed, with a nearly linear *I*–*V* relationship confirming Ohmic contacts between the Ge spheres and Pt wires. The n‐type Ge spheres (doping concentration ∼10^17^ cm^−3^) functioned as photosensitive elements, exhibiting increased electrical conductivity under illumination, evidenced by a change in the *I*–*V* curve compared with dark conditions. While the contrast between dark current and photocurrent was limited (potentially due to heavy doping facilitating Ohmic contact with Pt), this approach dramatically increased the density of functional components per fiber length compared to continuous‐core photosensitive fibers [53].

### 5.2 Semiconductor p‐n Junction

The realization of p‐n homo‐ and heterojunctions can significantly improve the efficiency and speed of in‐fiber‐integrated optoelectronic devices. Thermal drawing combined with laser treatment has proven to be a feasible route for obtaining size tunable dual‐sphere p‐n homo‐ and heterojunction particles via in‐fiber manipulation. The fabrication of such heterojunctions is inherently challenging due to material property disparities (e.g., viscosity, melting point, and surface tension), which result in disparate breakup periods and prevent formation via conventional contact‐by‐breakup methods. To bridge this material gap, the universal nature of laser‐induced thermocapillary convection was utilized. A Si/Ge dual‐core fiber was fabricated (Figure 8f), with the continuous cores subsequently transformed into distinct particle chains. Precise CO_2_ laser heating was then employed to generate centrosymmetric thermocapillary flow. This flow selectively drew a p‐Ge particle and an n‐Si particle into contact at the heating point, forming a stable heterojunction without coalescence, as experimentally demonstrated (Figure 8g). Successful electronic functionality of the released Ge/Si junction was confirmed by its rectifying *I*–*V* characteristics (Figure 8h) [54].

Crucially, this convection‐based manipulation enables the programmable assembly of sophisticated in‐fiber structures by joining diverse components, thereby overcoming intrinsic material limitations. Significantly, both particle generation and functional structure formation are accomplished within a single laser‐thermal step via adjustment of the heated area. This directed physical self‐assembly disrupts the fiber's axially invariant topology, enabling the simultaneous creation of periodically spaced, discrete electrical contacts between crystalline semiconductors and metals. This integration facilitates optoelectronic multisensory devices within the silica fiber. Furthermore, the selective breakup process dramatically increases component density per unit length, transforming a single cylindrical device into a linear array of electrically addressable spherical devices immobilized within the cladding.

## 6 Distilled Challenges and Perspectives

(1) While materials such as silicon‐germanium alloys and metal sulfides have been successfully integrated into fiber structures, the incorporation of next‐generation semiconductors including halide perovskites offers even greater promise. These emerging materials exhibit outstanding optoelectronic properties, including tunable bandgaps, high absorption coefficients, long carrier diffusion lengths, and low exciton binding energies, making them highly attractive for developing multifunctional and high‐performance semiconductor fiber devices. In addition, their compatibility with low‐cost, solution‐based fabrication techniques further enhances their potential for scalable integration. However, despite these advantages, significant technical challenges remain in realizing stable and uniform incorporation of such materials into fiber architectures. Addressing these issues will be crucial for advancing the field and unlocking the full potential of perovskite‐based optoelectronic fibers.

(2) There is a lack of effective methods for controlling the formation of high‐quality semiconductor interface junctions. Achieving stable, uniform incorporation of semiconductor junctions into fiber geometries requires overcoming significant obstacles related to multimaterial phase stability, crystallization control, and interfacial engineering under confined and dynamic fiber processing conditions. Future research should therefore prioritize developing robust processing strategies and fiber architectures that can accommodate a wider range of inorganic semiconductors, ultimately paving the way toward next‐generation, multifunctional optoelectronic fiber systems.

(3) The current strategies for regulating the geometric structure of semiconductor optoelectronic fibers remain limited. Most existing fiber fabrication techniques are designed to maintain axial continuity, aiming for uniform and consistent distribution of multiple materials along the fiber length. However, this structural uniformity inherently conflicts with the design requirements of modern optoelectronic devices, which often rely on the integration of multiple, spatially discrete junctions with varying functionalities. Although emerging approaches, such as those demonstrated in thermal drawing technology, have shown the potential to break axial continuity and introduce localized functional regions, these techniques are still in their infancy. Future research should focus on developing versatile and scalable methods for geometric modulation within fibers, such as localized doping, segmentation, or patterned deposition, to enable spatially programmable architectures. Such advancements would unlock new possibilities for multifunctional integration, device miniaturization, and reconfigurable fiber‐based optoelectronic systems.

## 7 Conclusions

In this review, we have outlined recent advances in the engineering of semiconductor optoelectronic fibers across three key dimensions: semiconductor material selection and processing, junction architecture design, and structural geometry optimization. We began by introducing the fundamental types of semiconductor junctions and the operating principles of representative fiber‐integrated optoelectronic devices. We then explored strategies for controlling the morphology and crystallization behavior of semiconductors within fibers, as well as methods for constructing well‐defined in‐fiber junctions and interfaces with increasingly complex layouts. Finally, we identified the major challenges that remain, including material compatibility, process scalability, and device integration stability. Looking ahead, further development will rely on interdisciplinary efforts to unify materials science, device physics, and fiber processing technologies. Addressing these challenges will be essential for unlocking the full potential of semiconductor optoelectronic fibers in next‐generation wearable systems, intelligent fabrics, and distributed sensing networks.

## Conflicts of Interest

The authors declare no conflicts of interest.

## Data Availability

Data sharing not applicable to this article as no datasets were generated or analysed during the current study.
